# Mechanisms of the Anti-Obesity Effects of Oxytocin in Diet-Induced Obese Rats

**DOI:** 10.1371/journal.pone.0025565

**Published:** 2011-09-27

**Authors:** Nicolas Deblon, Christelle Veyrat-Durebex, Lucie Bourgoin, Aurélie Caillon, Anne-Lise Bussier, Stefania Petrosino, Fabiana Piscitelli, Jean-Jacques Legros, Vincent Geenen, Michelangelo Foti, Walter Wahli, Vincenzo Di Marzo, Françoise Rohner-Jeanrenaud

**Affiliations:** 1 Division of Endocrinology, Diabetology and Nutrition, Department of Internal Medicine, Faculty of Medicine, University of Geneva, Geneva, Switzerland; 2 Department of Cell Physiology and Metabolism, Faculty of Medicine, University of Geneva, Geneva, Switzerland; 3 Endocannabinoid Research Group, Institute of Biomolecular Chemistry, CNR, Naples, Italy; 4 Centre Hospitalier Régional de la Citadelle, University of Liege, Liege, Belgium; 5 Centre of Immunology, University of Liege, CHU B-23, Liege, Belgium; 6 Center for Integrative Genomics, National Research Center Frontiers in Genetics, University of Lausanne, Lausanne, Switzerland; Clermont Université, France

## Abstract

Apart from its role during labor and lactation, oxytocin is involved in several other functions. Interestingly, oxytocin- and oxytocin receptor-deficient mice develop late-onset obesity with normal food intake, suggesting that the hormone might exert a series of beneficial metabolic effects. This was recently confirmed by data showing that central oxytocin infusion causes weight loss in diet-induced obese mice. The aim of the present study was to unravel the mechanisms underlying such beneficial effects of oxytocin. Chronic central oxytocin infusion was carried out in high fat diet-induced obese rats. Its impact on body weight, lipid metabolism and insulin sensitivity was determined. We observed a dose-dependent decrease in body weight gain, increased adipose tissue lipolysis and fatty acid β-oxidation, as well as reduced glucose intolerance and insulin resistance. The additional observation that plasma oxytocin levels increased upon central infusion suggested that the hormone might affect adipose tissue metabolism by direct action. This was demonstrated using *in vitro*, *ex vivo*, as well as *in vivo* experiments. With regard to its mechanism of action in adipose tissue, oxytocin increased the expression of stearoyl-coenzyme A desaturase 1, as well as the tissue content of the phospholipid precursor, N-oleoyl-phosphatidylethanolamine, the biosynthetic precursor of the oleic acid-derived PPAR-alpha activator, oleoylethanolamide. Because PPAR-alpha regulates fatty acid β-oxidation, we hypothesized that this transcription factor might mediate the oxytocin effects. This was substantiated by the observation that, in contrast to its effects in wild-type mice, oxytocin infusion failed to induce weight loss and fat oxidation in PPAR-alpha-deficient animals. Altogether, these results suggest that oxytocin administration could represent a promising therapeutic approach for the treatment of human obesity and type 2 diabetes.

## Introduction

The neurohypophysial hormone, oxytocin (OT), is a nonapeptide synthesized both centrally and peripherally. Within the central nervous system, the OT gene is expressed in neurons of the hypothalamic paraventricular (PVN) and supraoptic nuclei. The magnocellular OT neurons in these nuclei project to the neurohypophysis and are the major source of systemically released OT, whereas parvocellular OT neurons of the PVN project centrally. OT is also synthesized peripherally in several organs, including the ovary, testis, thymus, kidney, and heart [1]. To date, a single OT receptor (*OTR*) has been cloned. It is expressed in various tissues, including adipose tissue [1]. Consistent with its wide distribution of production and binding sites, OT has been implicated in several central and peripheral processes [1]. Amongst these, acute peripheral or central OT injections were shown to decrease food intake in rats [2]. Interestingly, in patients with the Prader-Willi syndrome, characterized by extreme hyperphagia leading to morbid obesity, a reduced number of OT neurons and a smaller volume of the PVN-containing OT-expressing neurons were reported [3]. Recent evidence also suggests that OT might be involved in the regulation of metabolic homeostasis. Thus, it was demonstrated that mice deficient in either *OT* or *OTR* developed late onset obesity despite normal food intake [4], [5]. Additionally, plasma OT levels were reported to be decreased in diet-induced obese mice and increased in synaptotagmin-4 deficient mice that are protected against diet induced obesity [6]. Finally, central OT infusion was shown to cause body weight loss in diet-induced obese mice [6]. However, the mechanisms underlying such beneficial effects of central OT infusion on metabolic homeostasis have not been unraveled so far and their understanding was therefore the first aim of the present study. Our results revealed that central OT infusion causes weight loss in diet-induced obese rats by increasing adipose tissue lipolysis and fatty acid β-oxidation via the production of oleoylethanolamide, a PPAR-alpha activator [7]. Our second aim was to delineate the pathways involved in such central OT effects. It should be recalled at this point that OT is one of the few hormones known to induce its own synthesis, as well as central and peripheral release [1]. This occurs in physiological situations, such as lactation and labor, during which OT is synthesized centrally and released into the bloodstream to act on its target tissues, namely the mammary gland and the uterus [1]. Data in the literature also indicate that OT exerts direct actions on human multipotent adipose-derived stem (hMADS) cells, as well as in isolated adipocytes, although the latter results depended on the OT dose used [8], [9]. Due to the above-mentioned considerations, the possibility that centrally infused OT might influence adipose tissue metabolism by direct peripheral action was tested and demonstrated using three different experimental approaches. Finally, we strengthened the hypothesis that OT might influence body weight gain and adipose tissue metabolism via PPAR-alpha by testing its effects in PPAR-alpha knockout mice.

## Materials and Methods

### Ethics statement

All procedures were performed in accordance with the Institutional Ethical Committee of Animal Care in Geneva and Cantonal Veterinary Office. The Institutional Ethical Committee of Animal Care in Geneva and Cantonal Veterinary Office approved this study through experimentation ID: 1034/3025/2-R.

### Animals

Male Wistar rats (300–325 g) were fed a 45% high fat diet (HFD) (Ssniff® EF R/M acc. D12451 (I) mod., ssniff Spezialdiäten GmbH, Soest, Germany) for 7 weeks to induce obesity. PPAR-alpha knockout [10] and wild-type mice received normal mice chow.

### Treatments

Centrally infused HFD-induced obese rats were intracerebroventricularly (i.c.v.) infused with saline or OT (NeoMPS®, Strasbourg, France) delivered continuously at doses of 1.6 nmol/d or 16 nmol/d over 14 days using osmotic minipumps (Alzet®, model 2001, Alza Corporation, Cupertino, CA), as previously described [11]. These doses of OT were chosen on the basis of the literature [2], as well as on preliminary acute experiments carried out before starting this project. Peripherally infused lean and HFD-induced obese rats received subcutaneous (s.c.) infusions of saline or OT at 50 nmol/d for 14 days using osmotic minipumps. Pair-fed controls were included when OT infusions reduced food intake. PPAR-alpha knockout and wild-type mice were subcutaneously infused with saline or OT at 50 nmol/day for 3 days using osmotic minipumps.

### Feeding pattern parameters, respiratory exchange ratio and locomotor activity

Analyzes were performed at the end of the 2 week central infusion using the 12-cage LabMaster system (TSE Systems GmbH, Berlin, Germany) of the Small Animal Phenotyping Core Facility (CMU, University of Geneva, Geneva), under controlled temperature (22±1°C) and lighting (12 h light-dark cycle). The LabMaster consists in a combination of highly sensitive feeding and drinking sensors for automated online measurements. Before recording, animals were allowed a 4-day acclimatization period in training cages.

### Plasma measurements

Plasma glucose was measured with the glucose oxidase method (Glu, Roche Diagnostics GmbH, Rotkreuz, Switzerland). Plasma nonesterified fatty acid (FFA), glycerol and triglyceride (TG) levels were determined with commercial kits (NEFA C: Wako Chemicals, GmbH, Neuss, Germany; Glycerol: Free Glycerol Reagent, Sigma, Switzerland and TG enzymatic PAP150: Biomérieux, Marcy l'Etoile, France). Plasma OT levels were determined with a home-made ELISA [12]. Measurements were performed at the end of the 14-day treatment periods.

### Glucose tolerance tests

Glucose (1.5 g/kg body wt) was intraperitoneally administered 4 h after food removal. Blood glucose levels were measured from tail blood samples collected at 0, 15, 30, 60, 90, and 120 min after glucose injection.

### Euglycemic-hyperinsulinemic clamps

Global glucose utilization rates were measured with euglycemic-hyperinsulinemic clamps [13].

### Body composition

When OT was delivered peripherally in rats, an EchoMRI-700 quantitative nuclear magnetic resonance analyzer (Echo Medical Systems, Houston, TX) was used to measure total fat mass and lean body mass at the beginning and the end of treatments (days 0 and 10).

### Primer sequences

Primers were designed with PrimerExpress software (http://phym.unige.ch/) (Table S1). Results were normalized to the expression levels of ribosomal protein S29.

### Endocannabinoids

Endocannabinoids were measured as previously described [14].

### In vitro experiments

Murine 3T3-L1 fibroblasts [15] were cultured in Dulbecco's modified eagle medium (DMEM) with 4.5 g/l glucose supplemented with 10% heat-inactivated depleted calf serum at 37°C, 8% CO_2_. They were induced to differentiate into adipocytes at two days post confluence as described in [16]. Differentiation was assumed when over 90% of cells showed large lipid droplets in the cytoplasm. After differentiation, 3T3-L1 adipocytes were incubated with saline and 5 µM OT during 24 h.

### Lipolysis assay

Epididymal fat pads from lean Wistar rats were incubated at 37°C in the presence of Krebs-Ringer-Hepes buffer containing 2% FA-free BSA and 0.1% glucose. After 4 h of incubation in the presence of either saline or OT (10 nM), the amount of glycerol and free fatty acid released in the medium were determined with commercial kits (Glycerol: Free Glycerol Reagent, Sigma, Switzerland; NEFA C: Wako Chemicals, GmbH, Neuss, Germany).

### Statistics

Results are expressed as means ± SEM. For experiments including two sets of data, comparisons were performed with parametric (Student's t test) and non-parametric (Mann-Whitney test) tests when normality and equal variance failed. Experiments with more than two groups were analyzed using ANOVA, followed by the post-hoc Bonferroni test. Statistical significance was established at *P<0.05, **P<0.01, ***P<0.005.

## Results

### OT infusion decreases body weight gain

**Figure 1:**
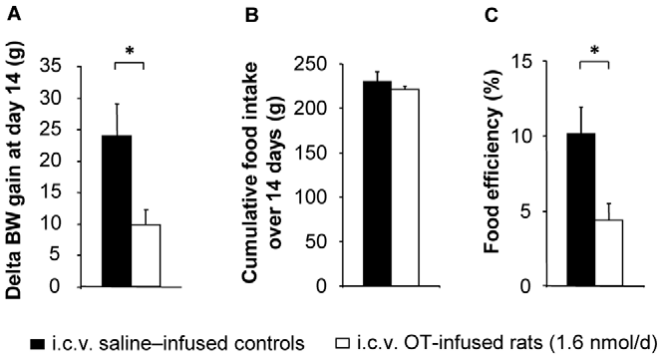
Central OT infusion causes body weight loss independently from changes in food intake. The measurements were performed over a 14-day experimental period (weeks 5 through 7 of a high fat diet): (A) Cumulative body weight changes; (B) cumulative food intake; (C) food efficiency ((body weight gain/cumulative food intake over the 2 week experimental period) x 100). Filled bars: i.c.v. saline–infused controls; open bars: i.c.v. OT-infused rats (1.6 nmol/d). Values are mean ± SEM of 6 to 7 rats/group. *P<0.05 compared to controls.

To study the role of OT on metabolic homeostasis, rats were fed a high fat diet (HFD, 45% fat) for 7 weeks to induce obesity. During the last 2 weeks of diet-induced obesity, the animals were intracerebroventricularly (i.c.v.) infused with either OT (1.6 nmol/d) or saline using osmotic minipumps. Chronic i.c.v. OT infusion lowered body weight gain, resulting in more than a 50% decrease in cumulative body weight gain compared with the effect of saline (Figure 1A; *P*<0.05). This occurred without any change in food intake (Figure 1B) or in meal patterns (Table S2), therefore leading to a marked decrease in food efficiency (Figure 1C; *P*<0.05).

### OT infusion stimulates lipid metabolism

**Figure 2:**
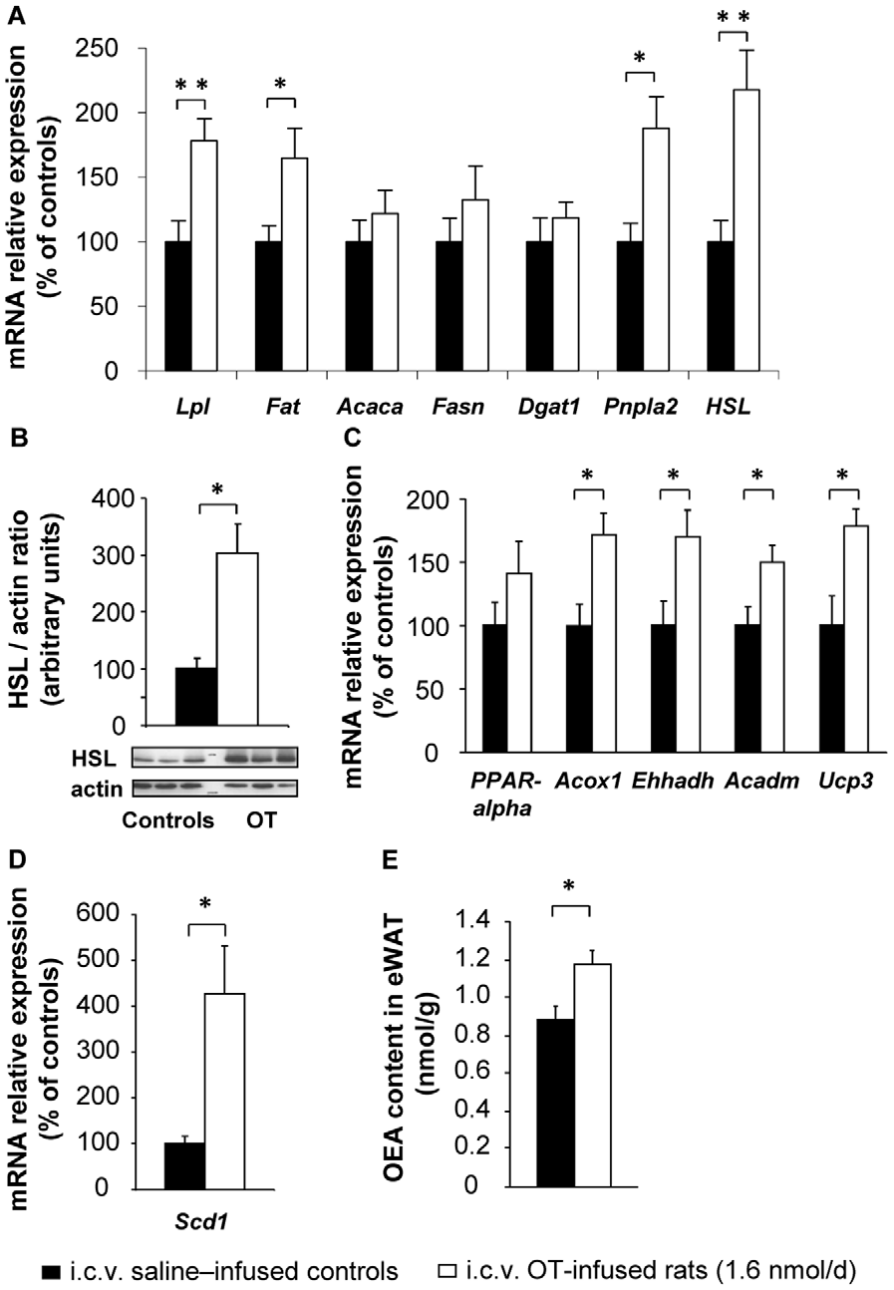
Central OT infusion stimulates lipid metabolism. The following analyses were performed on epididymal white adipose tissue (eWAT) of i.c.v. saline–infused controls (filled bars) and i.c.v. OT-infused rats (1.6 nmol/d; open bars): (A) mRNA expression of enzymes related to lipid metabolism; (B) Western blot analysis of HSL standardized to actin expression; (C) mRNA expression of PPAR-alpha and PPAR-alpha target genes; (D) Scd1 mRNA expression; (E) Oleoylethanolamide (OEA) content in eWAT. Values are mean ± SEM of 6 to 7 rats/group. *P<0.05, **P<0.01 compared to controls.

To determine whether OT modulated peripheral metabolism, we determined plasma glucose, insulin, leptin, free fatty acid (FFA), glycerol and triglyceride (TG) levels. Of these parameters, only glycerol and TG concentrations were affected by central OT infusion, the former being increased, while the latter were reduced (Table 1; *P*<0.05). To delineate the mechanisms responsible for the decrease in TG levels, we analyzed the expression of enzymes involved in lipid metabolism in adipose tissue. This is indeed an important tissue for lipid metabolism, in which the *OTR* appears to be expressed, as determined in primary and cultured adipocytes [17], [18], [19]. We focused on epididymal white adipose tissue (eWAT), as it is considered an intra-abdominal fat depot with high metabolic relevance. Central OT infusion promoted an increase in the eWAT mRNA expression of lipoprotein lipase (*Lpl*) (Figure 2A; *P*<0.01) and fatty acid transporter (*Fat*, also known as CD36) (Figure 2A; *P*<0.05), two enzymes responsible for the uptake of circulating TG and fatty acids, respectively. It did not modify the expression of enzymes involved in lipogenesis and TG storage; for example, acetyl-coenzyme A carboxylase alpha (*Acaca*, also known as ACC-*alpha*), fatty acid synthase (*Fasn*), and diacylglycerol O-acyltransferase homolog 1 (*Dgat1*). However, central OT infusion increased the mRNA levels of two enzymes involved in lipolysis, namely patatin-like phospholipase domain containing 2 (*Pnpla2*) (*P*<0.05) and hormone-sensitive lipase (*HSL*) (P<0.01). The stimulatory effect of OT on HSL was also detected at the protein level (Figure 2B; *P*<0.05).

**Table 1:** Effects of i.c.v. oxytocin (1.6 nmol/d) infusion on plasma glucose, insulin, leptin, FFA, glycerol, TG, oleoylethanolamide (OEA), palmitoylethanolamide (PEA), anandamide (AEA) and 2-arachidonoylglycerol (2-AG) levels.

	Saline-infused rats	OT-infused rats
**Glucose (mg/dl)**	159.1±5.7	159.5±4.1
**Insulin (ng/ml)**	2.5±0.7	1.7±0.3
**Leptin (ng/ml)**	13.9±3.7	11.3±2.2
**FFA (mmol/l)**	0.82±0.06	0.70±0.06
**Glycerol (µg/ml)**	50.6±5.1	63.6±3.2 *
**TG (mmol/l)**	1.11±0.09	0.80±0.05 *
**OEA (pmol/ml)**	145±13	178±15
**PEA(nmol/ml)**	1.34±0.16	1.63±0.15
**AEA (pmol/ml)**	18±2.9	19±2.3
**2-AG (pmol/ml)**	78±13	53±4.9

Values are mean ± SEM of 6–7 animals per group. * P<0.05 versus saline-infused controls. P = NS for all other comparisons.

Due to the increases in TG uptake and in lipolysis, we expected enhanced intracellular availability of FFA in adipocytes, as well as elevated circulating FFA levels in OT-infused rats compared to controls. However, the plasma FFA concentrations were unaltered by central OT infusion (Table 1). This suggested that FFA utilization had increased. We therefore measured the mRNA expression of enzymes involved in fatty acid β-oxidation. We found that OT infusion increased the eWAT expression of acyl-CoA oxidase 1 (*Acox1*) (Figure 2C; *P*<0.05), enoyl-CoA hydratase/3-hydroxyacyl-CoA dehydrogenase (*Ehhadh*, also known as *HD*) (*P*<0.05), medium chain acyl-CoA dehydrogenase (*Acadm*, also known as *MCAD*) (*P*<0.05), and uncoupling protein 3 (*Ucp3*) (*P*<0.05). However, we did not observe any change in the expression of *PPAR-alpha*, a ligand-activated transcription factor that regulates the expression of the genes encoding the above-mentioned proteins.

To determine whether these results were specific for adipose tissue, we evaluated the mRNA expression of most of these lipid metabolism-related genes in skeletal muscles and in the liver. We observed that central OT infusion did not modify the expression of these genes in quadriceps and in the liver (Figure S1), a tissue in which the OT receptor expression is suppressed by CpG island methylation in the gene promoter region [20]. These results suggest that the OT anti-obesity effects are primarily exerted at the level of adipose tissue.

### OT infusion increases oleoylethanolamide production in adipose tissue

We then attempted to unravel the mechanisms responsible for the OT-induced stimulatory effect on adipocyte fatty acid β-oxidation. As shown in Figure 2D, central OT infusion promoted a marked increase in the mRNA expression of stearoyl-coenzyme A desaturase 1 (*Scd1*; Figure 2D; *P*<0.05). Scd1 converts the saturated fatty acids, palmitic acid (16∶0) and stearic acid (18∶0) into the monounsaturated fatty acids, palmitoleic acid (16∶1) and oleic acid (18∶1), respectively. The resulting fatty acids can either be incorporated into the cell membrane as phospholipids (PL) or stored as TG [21]. Because we did not observe any changes in the mRNA expression of enzymes involved in TG storage in the eWAT of OT-infused animals (Figure 2A), we hypothesized that OT might have increased palmitoleic and oleic acid synthesis and their incorporation as PL into the cell membrane. We further reasoned that oleic acid can be transferred from the sn-1 position of phosphatidylcholine (PC) to the free amine of phosphatidylethanolamine (PE) in order to form N-oleoyl-phosphatidylethanolamine (NOPE) [7]. Then, NOPE is cleaved by N-acyl-phosphatidylethanolamine phospholipase D (NAPE-PLD) to release oleoylethanolamide (OEA), a member of the *N*-acylethanolamine family. Therefore, we could infer changes in membrane PL incorporation by measuring changes in OEA production in adipose tissue. We found that, compared to saline infusion, chronic i.c.v. OT administration increased the OEA content in eWAT (Figure 2E; *P*<0.05). Of note, OT did not modify the eWAT content of other related lipids, including the saturated analog palmitoylethanolamide (PEA), derived from palmitic acid, or the endocannabinoids, anandamide (AEA) and 2-arachidonoylglycerol (2-AG), derived from arachidonic acid (data not shown), nor did it alter hepatic (data not shown), or plasma levels of OEA or other endocannabinoids (Table 1). Interestingly, OEA is an agonist of PPAR-alpha that was demonstrated to bind to the PPAR-alpha ligand-binding-domain (LBD), leading to the activation of PPAR-alpha transcriptional activity [22]. Moreover, OEA has been shown to stimulate lipolysis and fatty acid oxidation, effects resulting from an OEA-induced activation of PPAR-alpha [23], [24]. Thus, the observation that OT increased OEA synthesis and PPAR-alpha target gene expression suggests that although OT does not increase PPAR-alpha expression, it stimulates its transcriptional activity by increasing eWAT levels of the PPAR-alpha ligand, OEA.

### Dose-dependency of the effect of OT infusion

**Figure 3:**
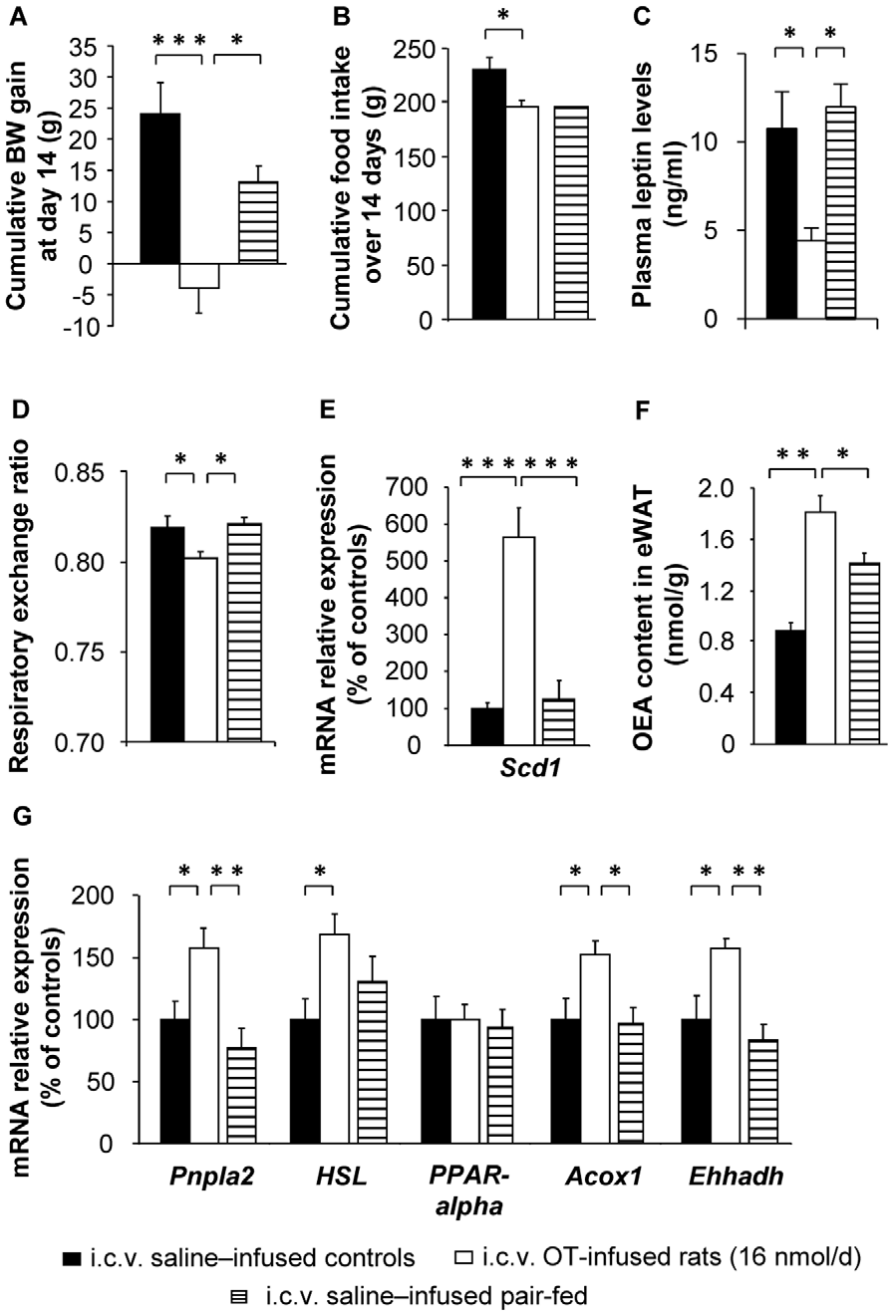
Dose-dependency of the effect of central OT infusion. After 14-day treatments (weeks 5 to 7 of high fat diet, 45% fat), the following measurements were made in i.c.v. saline–infused controls (filled bars), i.c.v. OT-infused rats (16 nmol/d; open bars), and i.c.v. saline-infused pair-fed (PF) controls (hatched bars): (A) Delta body weight gain; (B) cumulative food intake; (C) plasma leptin levels; (D) respiratory exchange ratio (VO_2_: VCO_2_); (E) *Scd1* mRNA expression; (F) OEA content in epididymal white adipose tissue (eWAT); and (G) mRNA expression of lipid metabolism-related enzymes in eWAT. Values are mean ± SEM of 6 to 7 animals/group. *P<0.05, **P<0.01, ***P<0.005, compared to controls.

Knowing that OEA is an agonist of PPAR-alpha [7], we postulated that it may mediate the stimulatory effects of OT on lipid oxidation in adipose tissue. To strengthen this hypothesis, we first examined the dose-dependency of these effects. HFD-induced obese rats were i.c.v. infused for 14 days with a ten-fold higher dose of OT (16 nmol/d), using osmotic minipumps. As shown in Figure 3B, central OT infusion at this dose promoted a decrease in cumulative food intake compared to controls (*P*<0.05). Upon studying meal patterns at the end of the 2 week central OT or saline infusion, we observed a reduction in meal number (Table S3, *P*<0.05), without any change in meal size or duration. The satiety ratio (intermeal interval/meal size), an index of the satiety time produced by each gram of food consumed, was higher in OT- than in saline-infused animals (Table S3, *P*<0.05). Locomotor activity was unaltered by either the low (1.6 nmol/d) or the high dose (16 nmol/d) of central OT infusion (data not shown).

To determine the metabolic effects elicited by OT independently from changes in food intake, a saline-infused control group was pair-fed (PF) to match the consumption of OT-treated animals. Body weight gain (Figure 3A; *P*<0.005) as well as plasma leptin levels (Figure 3C; *P*<0.05) of OT-infused rats were decreased compared to the saline–infused and the PF control groups. The respiratory exchange ratio (RER), measured by indirect calorimetry, was also lower in OT- than in saline-infused animals (Figure 3D; *P*<0.05), independently from changes in food intake (Figure 3D; *P*<0.01). This indicated a lower rate of carbohydrate utilization and a higher rate of lipid oxidation, suggesting that OT-infused rats used more lipids as energy source than the two control groups. Of note, we observed that energy expenditure was unaltered by central OT infusion (data not shown). In addition, the high dose of OT stimulated *Scd1* mRNA expression and OEA eWAT content more markedly than the low dose (5.6- and 2.0-fold *versus* 4.2- and 1.3-fold stimulations, respectively). This further indicated that central OT action on lipid metabolism in adipose tissue involved OEA synthesis via Scd1, independent from changes in food intake (Figures 3E and F; *P*<0.05). Consistent with the results found with the ten-fold lower dose, the 16 nmol/d OT infusion did not modify the eWAT mRNA expression of *PPAR-alpha*, but resulted in food intake-independent increases in the expression of the PPAR-alpha target genes *Acox1* and *Ehhadh* (Figure 3G; *P*<0.05). It also stimulated the expression of the genes encoding the main lipolytic enzymes, *Pnpla2 and HSL* (*P*<0.05).

### Central OT infusion increases OT synthesis and release into the bloodstream

Knowing that OT itself can activate OT neurons, thereby inducing its own synthesis, as well as central and peripheral release, we next determined the impact of chronic i.c.v. OT infusion on hypothalamic *OT* mRNA expression, as well as circulating OT levels.

**Figure 4:**
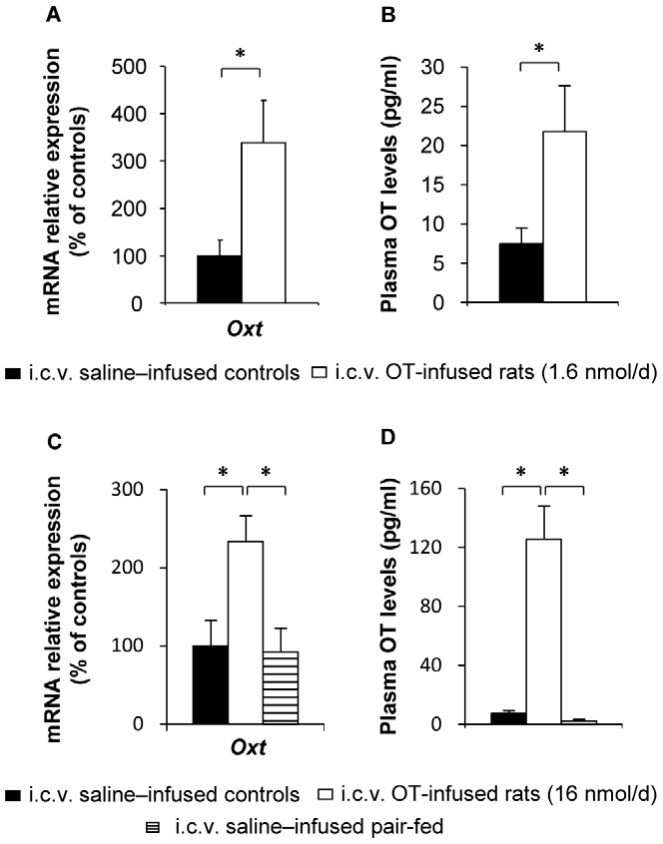
Central OT infusion induces hypothalamic OT synthesis and release into the bloodstream. The following parameters were measured at the end of 14-day treatments with two doses of i.c.v. OT infusion: (A) Oxytocin expression (*Oxt*) in rat hypothalamus; (B) plasma OT levels in saline–infused controls (filled bars) and OT-infused rats (1.6 nmol/d, open bars). Values are mean ± SEM of 6 to 7 rats/group. *P<0.05 compared to controls; (C) Oxytocin expression (*Oxt*) in rat hypothalamus; (D) plasma OT levels in saline–infused controls (filled bars), OT-infused rats (16 nmol/d, open bars) and pair-fed (PF) controls (hatched bars). Values are mean ± SEM of 6 to 7 rats/group. *P<0.05 compared to controls.

As shown by Figure 4, both doses of chronic i.c.v. OT infusion increased hypothalamic *OT* mRNA expression, as well as plasma OT levels. Moreover, while 1.6 nmol/d OT infusion led to a 3-fold increase in plasma OT levels (Figure 4B; *P*<0.05), the ten-fold higher dose (16 nmol/d) resulted in a 15-fold increase in these levels (Figure 4D; *P*<0.05). This occurred in the presence of similar changes in *OT* mRNA expression in response to the two doses, suggesting that OT dose-dependently affected its own release, rather than synthesis.

Of note, we observed that the eWAT mRNA expression of *OT* and *OTR* was unaltered by either the low (1.6 nmol/d) or the high dose (16 nmol/d) of central OT infusion (Figure S2).

Altogether, these observations suggested that OT might modulate lipid metabolism in adipose tissue by direct action.

### OT affects peripheral lipid metabolism by direct peripheral action

**Figure 5:**
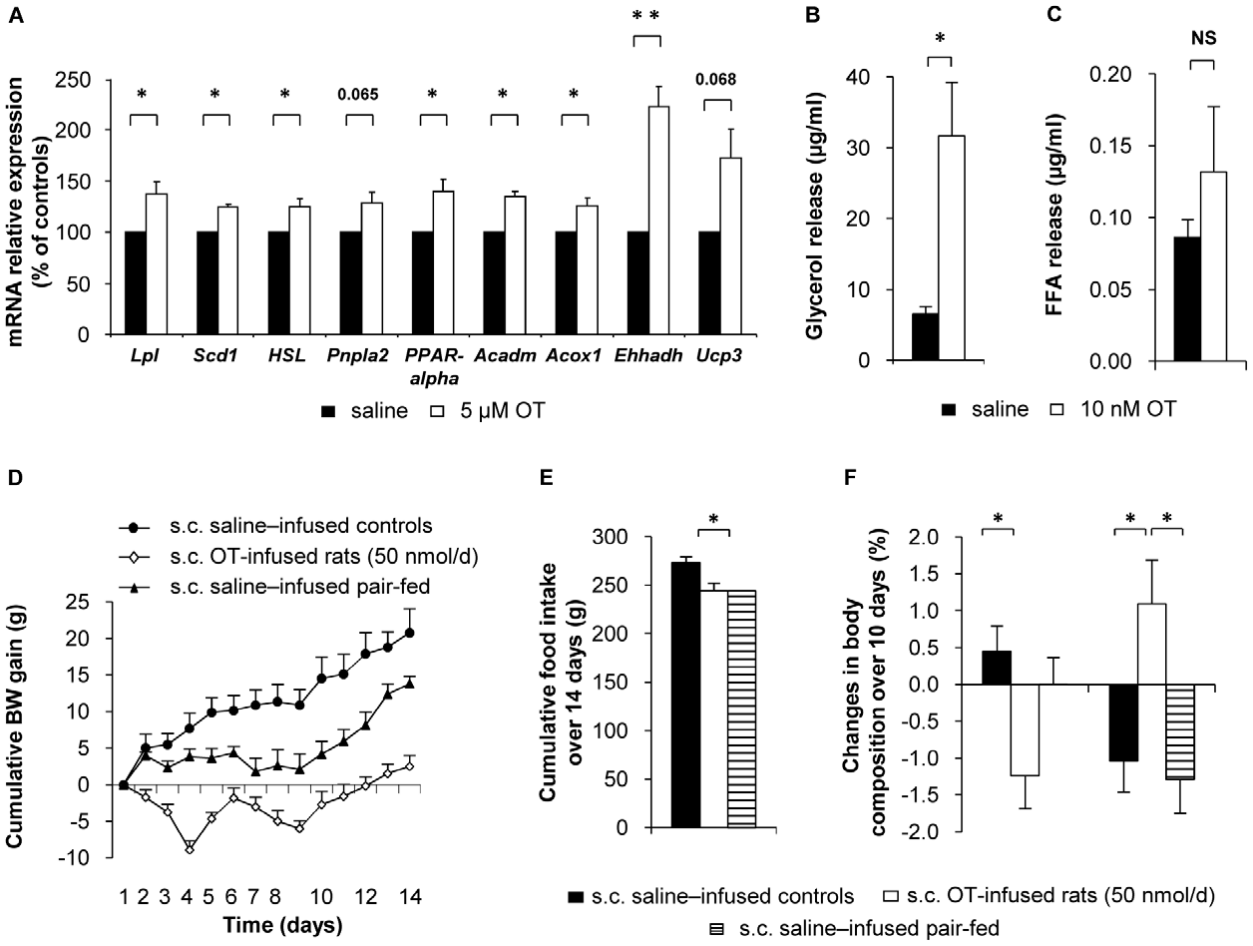
OT directly affects lipid metabolism. (A) Lipid metabolism-related enzyme expression in differentiated 3T3-L1 adipocytes (24 h saline or 5 µM OT). Values are mean ± SEM of three independent experiments. *P<0.05, **P<0.01 compared to controls. (B–C) Epididymal fat pads from lean Wistar rats were incubated at 37°C in the presence of Krebs-Ringer-Hepes buffer containing 2% FA-free BSA and 0.1% glucose. After 4 h of incubation in the presence of either saline or OT (10 nM), the amount of (B) glycerol and (C) free fatty acid released in the medium was measured. Values are mean ± SEM of three independent experiments. (D–F) Measurements performed over the 14-day s.c. saline or OT treatment in lean rats fed a standard diet: (D) Cumulative body weight gain; (E) cumulative food intake; (F) changes in body composition between days 0 and 10 of treatment. Saline–infused controls (black circles, filled bars), OT-infused rats (50 nmol/d; white diamonds, open bars), and saline-infused PF controls (black triangles, hatched bars). Values are mean ± SEM of 6 to 7 rats/group. *P<0.05.

To test the hypothesis that OT could affect peripheral lipid metabolism by direct peripheral action, we first assessed the effect of OT on lipid metabolism *in vitro* using differentiated 3T3-L1 adipocytes, known to express the OTR upon differentiation [19]. As shown in Figure 5A, OT increased the mRNA expression of all the lipid metabolism-related genes that were affected by *in vivo* central OT infusion. This included a stimulatory effect of OT on *PPAR-alpha*, which was not observed in WAT *in vivo*. Additionally, using the same in vitro system, we observed that OEA induced the mRNA expression of PPAR-alpha and of most of the PPAR-alpha target genes (Figure S3).

We next examined the effect of OT on lipolysis in *ex vivo* incubated epididymal fat pads from lean rats. As shown in Figure 5B, OT significantly increased glycerol release, suggesting increased adipose tissue lipolysis. In addition and in keeping with the results obtained *in vivo*, OT did not affect fatty acid release (Figure 5C), suggesting increased fatty acid β-oxidation.

Finally, we determined whether chronic peripheral OT infusion could affect lipid metabolism *in vivo*, in both lean and HFD-induced obese rats. Lean rats were peripherally infused with OT (50 nmol/d for 14 days, subcutaneous minipumps). At this dose, OT administration decreased food intake and body weight gain (Figures 5D and E; *P*<0.05). The impact of peripheral OT infusion on body composition was also evaluated using an EchoMRI-700 analyzer. We observed that OT promoted a significant decrease in fat mass and a concomitant increase in the percent lean body mass, independently from changes in food intake (Figure 5F; *P*<0.05).

**Figure 6:**
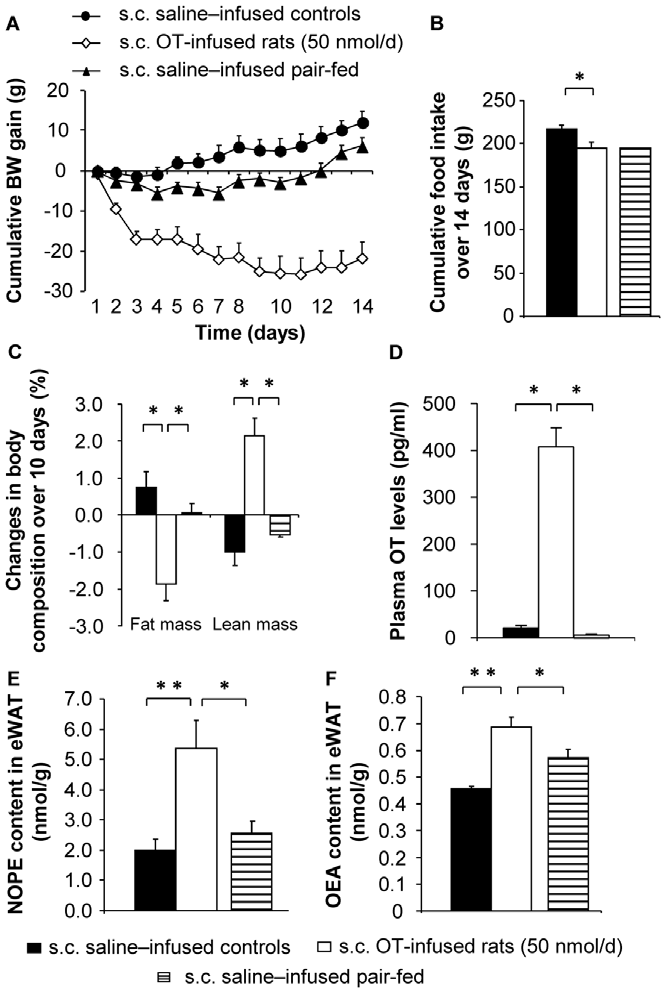
Peripheral OT effects in HFD fed rats. The measurements were performed over a 14-day experimental period (weeks 5 through 7 of a high fat diet) in s.c. saline–infused controls (black circles, filled bars), s.c. OT-infused rats (50 nmol/d; white diamonds, open bars), and s.c. saline-infused PF controls (black triangles, hatched bars): (A) Cumulative body weight gain; (B) cumulative food intake; (C) changes in body composition between days 0 and 10 of treatment. (D) Plasma OT levels; (E) NOPE and (F) OEA content in eWAT. Values are mean ± SEM of 7 to 8 rats/group. *P<0.05, **P<0.01 compared to controls.

Similar results, but more marked, were obtained by peripherally infusing OT in HFD-induced obese rats. Thus, when HFD-induced obese rats were peripherally infused with OT (50 nmol/d for 14 days, subcutaneous minipumps), the peptide decreased food intake and caused a strong body weight loss (Figures 6A and B; *P*<0.05). Such body weight loss was unrelated to the anorexigenic effect of the hormone, as it was not observed in the PF control group (Figure 6A). OT also promoted a significant decrease in fat mass and a concomitant increase in percent lean body mass, independently from changes in food intake (Figure 6C; *P*<0.05). Accordingly, we observed that OT decreased the eWAT TG, but did not change the FFA content (Figure S4). The OT-induced decrease in adipose tissue glycerol levels suggests that this metabolite is exported from the tissue into the bloodstream. Importantly in view of the potential direct OT action on adipose tissue, peripheral OT administration resulted in a 20-fold increase in plasma OT levels (Figure 6D; *P*<0.05). This was accompanied by increases in the eWAT content of OEA (Figure 6F, *P*<0.05), as well as its phospholipid precursor, N-oleoyl-phosphatidylethanolamine (NOPE) (Figure 6E, *P*<0.05) that were not mediated by the anorexigenic effect of OT.

### PPAR-alpha mediates direct peripheral OT action on lipid metabolism

**Figure 7:**
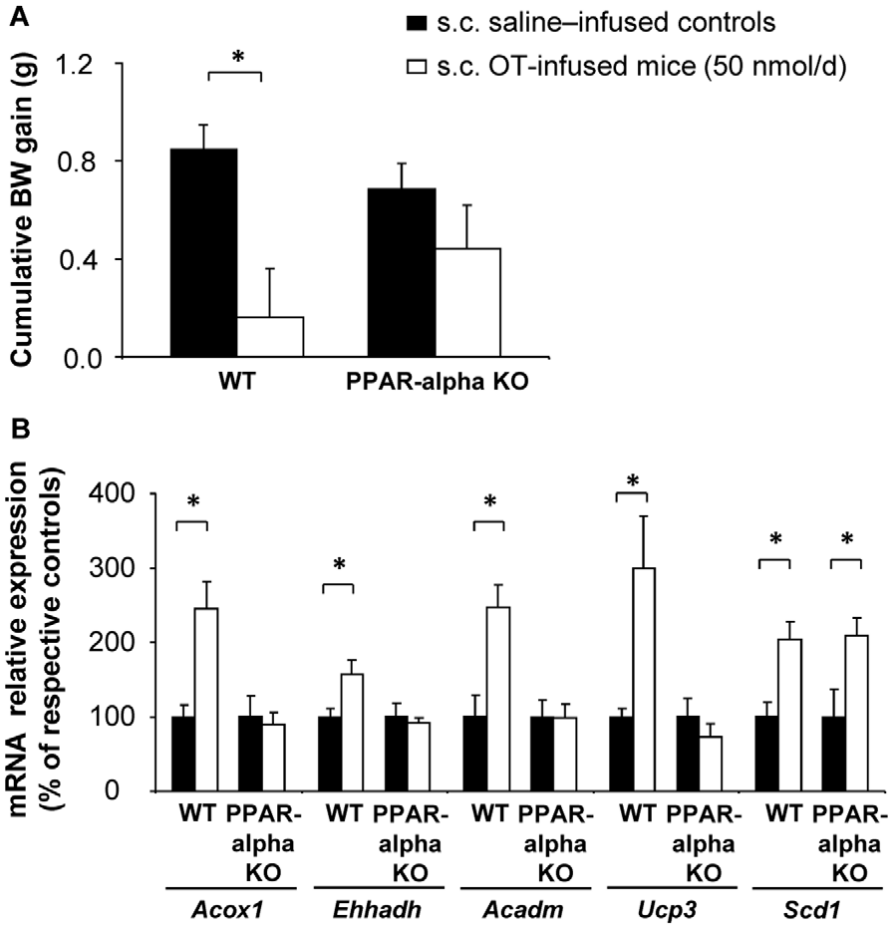
PPAR-alpha mediates peripheral OT effects. (A) Cumulative body weight gain after 3 days of s.c. saline or OT treatment in PPAR-alpha KO and wild-type (WT) mice. (B) eWAT mRNA expression of PPAR-alpha target genes and *Scd1* in PPAR-alpha KO and WT mice. Values are mean ± SEM of 5 animals/group. *P<0.05 compared to controls.

Based on our observation that OT infusion dose-dependently increased the adipose tissue content of the PPAR-alpha agonist, OEA, we reasoned that OEA might mediate the effects of OT through its action on PPAR-alpha transcriptional activity. To test this hypothesis, we infused OT peripherally in PPAR-alpha knockout (KO) and wild-type (WT) mice. As expected, the peripheral OT infusion induced a significant reduction in body weight gain in WT animals. However, it did not modify weight gain in PPAR-alpha KO mice (Figure 7A; *P*<0.05). Accordingly, the OT infusion promoted an increase in the mRNA expression of PPAR-alpha target genes in WT mice (*Acox1*, *Ehhadh*, *Acadm* and *Ucp3)*, but had no effect in the PPAR-alpha knockout animals (Figure 7B). Interestingly, *Scd1* expression similarly increased in both groups (Figure 7B, *P*<0.05), suggesting that the OT effect on *Scd1* was upstream PPAR-alpha activation.

### Both central and peripheral OT infusion reduces insulin resistance

**Figure 8:**
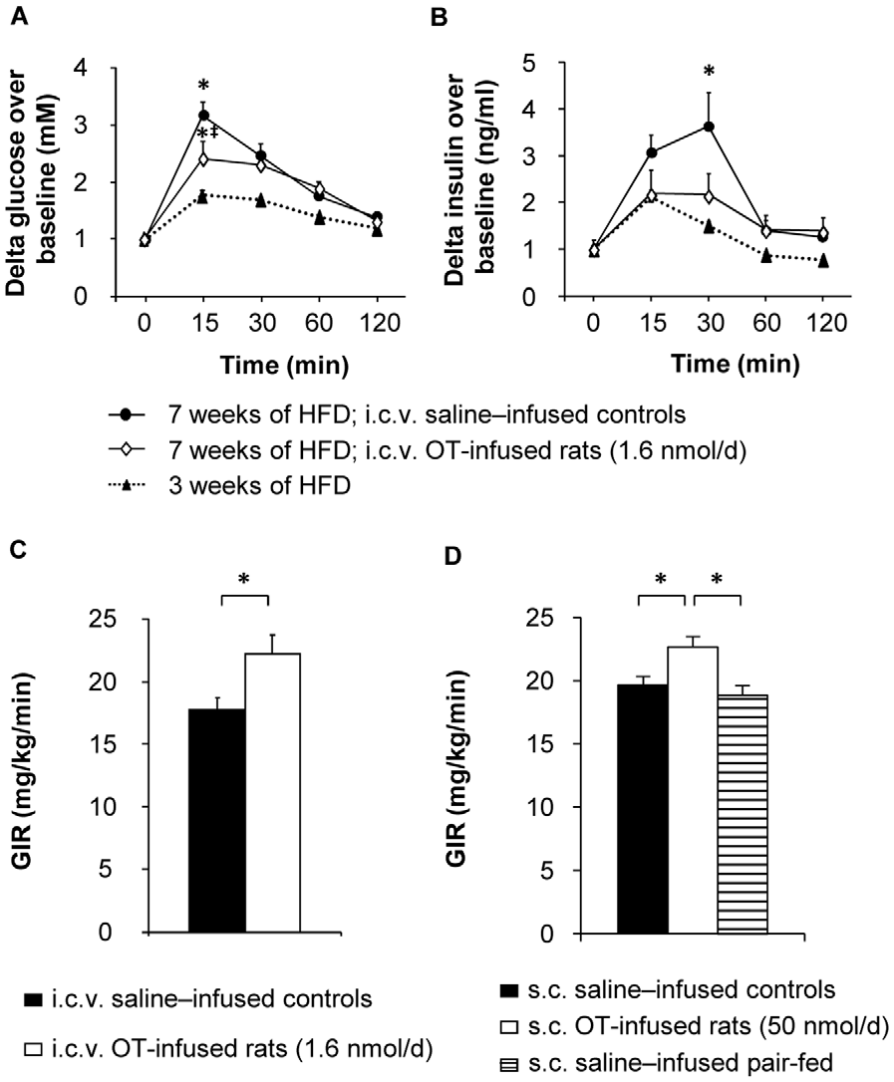
Central and peripheral OT infusion protects against high fat diet-induced insulin resistance. I.c.v. saline- (black circles) or OT- (1.6 nmol/d; white diamonds) infused rats received: glucose tolerance tests (1.5 g/kg) before (black triangles, dashed line; 3 weeks of HFD; n = 16 rats) or after infusions (7 weeks of HFD; 14-day i.c.v. infusions; n = 6 for each treatment group): (A) delta glucose and (B) delta insulin; One-way ANOVA: *P<0.05 compared to black triangles; ‡P<0.05 compared to black circles. (C–D) Euglycemic-hyperinsulinemic clamps performed at the end of 14-day treatments: (C) Glucose infusion rate (GIR) of i.c.v. saline- (filled bars) or OT- (1.6 nmol/d; open bars) infused rats. Values are mean ± SEM of 6 to 7 rats/group. *P<0.05 compared to controls. (D) GIR of s.c. saline–infused controls (filled bars), OT-infused rats (50 nmol/d; open bars), and saline-infused PF controls (hatched bars). Values are mean ± SEM of 6 to 7 rats/group. *P<0.05.

In view of the observations that OT-deficient mice, known to develop late-onset obesity, also exhibit decreased insulin sensitivity and impaired glucose tolerance [4], we determined whether chronic central OT infusion would affect these parameters in HFD-induced obese rats. We therefore performed glucose tolerance tests (GTT) and euglycemic-hyperinsulinemic clamps in i.c.v. saline- and OT-infused animals. Two GTTs were carried out: the first one after 3 weeks of HFD, and the second one after 7 weeks of HFD, at the end of the 2-week saline or OT infusion. We observed that, after 7 weeks of HFD, both saline- and OT-infused rats exhibited a deterioration in the second GTT compared to the first one (Figure 8A; *P*<0.05). However, this deterioration was significantly lower in the OT- than in the saline-infused group (Figure 8A; *P*<0.05). In addition, while it was accompanied by insulin oversecretion in saline-infused animals (Figure 8B; *P*<0.05), this did not occur in OT-infused rats, suggesting that OT might protect against HFD-induced glucose intolerance and insulin resistance.

To confirm these results, we performed euglycemic-hyperinsulinemic clamps. We found that, compared to saline-infused rats, i.c.v. OT-infused animals displayed significant increases in the glucose infusion rate (GIR; Figure 8C; *P*<0.05), indicating the presence of increased insulin sensitivity. Similar results were obtained by infusing OT peripherally (Figure 8D; *P*<0.05). The latter data showed in addition that the OT-induced increase in insulin sensitivity was unrelated to the decrease in food intake, as it was not observed in the PF control group.

**Figure 9:**
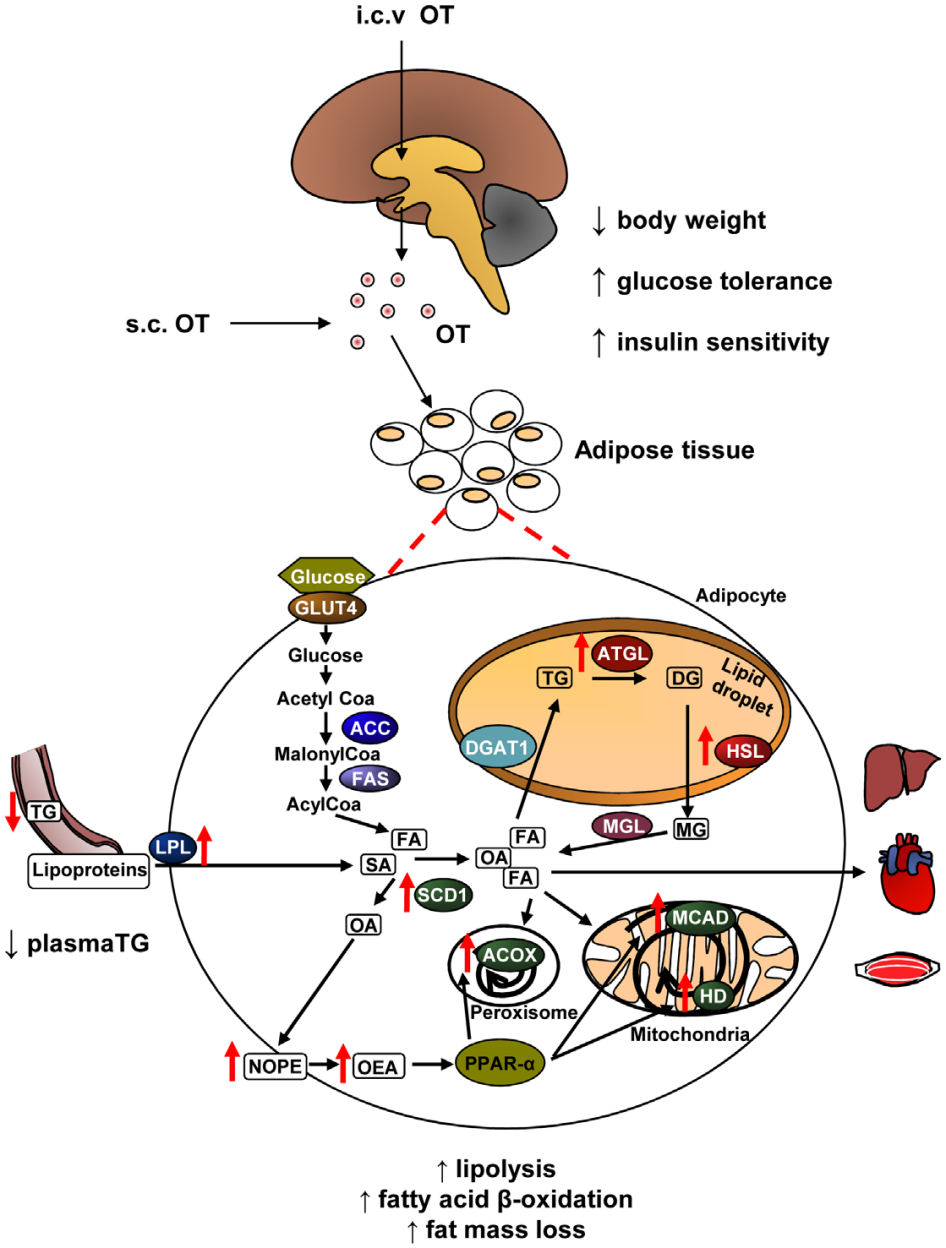
Summary of the metabolic effects of oxytocin. Upon chronic central (i.c.v.) or peripheral (s.c.) infusion into diet-induced obese rats, oxytocin (OT) increases triglyceride (TG) uptake, lipolysis, and fatty acid β-oxidation in adipose tissue. OT activates stearoyl-Coenzyme A desaturase 1 (Scd1) to produce the endocannabinoid oleoylethanolamide (OEA), a known ligand of PPAR-alpha. The action of OT on fatty acid β-oxidation is thus exerted by direct activation of PPAR-alpha target genes via the production of OEA. Red arrows indicate the direction (up or down) of regulation. Abbreviations: ACOX (acyl-CoA oxidase 1), ACC (acetyl-coenzyme A carboxylase alpha), ATGL (patatin-like phospholipase domain containing 2), DG (diglycerides), DGAT1 (diacylglycerol O-acyltransferase homolog 1), FA (fatty acid), FAS (fatty acid synthase), GLUT4 (glucose transporter-4), HD (enoyl-CoA hydratase/3-hydroxyacyl-CoA dehydrogenase), HSL (hormone-sensitive lipase), LPL (lipoprotein lipase), MCAD (medium chain acyl-CoA dehydrogenase), MG (monoglycerides), MGL (monoglyceride lipase), NOPE (N-oleoyl-phosphatidylethanolamine), OA (oleic acid), SA (stearic acid), PPAR-α (peroxisome proliferator-activator receptor-alpha).

The effects of OT on lipid metabolism in adipose tissue and their main consequences on metabolic homeostasis are summarized by Figure 9.

## Discussion

As mentioned in the Introduction, recent data showed that central OT infusion causes body weight loss in diet-induced obese mice [6]. The present study aimed at unraveling the mechanisms underlying such anti-obesity effects of OT. Our results first extended those of the literature by showing that central OT infusion decreased body weight gain in diet-induced obese rats independently from changes in food intake. Central OT administration also decreased plasma TG levels. These changes were accompanied by increases in the epididymal adipose tissue (eWAT) expression of *Lpl* and of enzymes involved in lipolysis and fatty acid β-oxidation, without any alteration in lipid metabolism in skeletal muscles or the liver. Although was not investigated in the present study, it is possible that, in addition to its effects on lipolysis and β-oxidation, OT could have modified adipogenesis. Indeed, it has been shown that OT and carbetocin (a stable OT analogue) negatively modulate adipogenesis, while promoting osteogenesis in human multipotent adipose-derived stem (hMADS) cells [8]. An important additional observation was that upon central infusion, hypothalamic mRNA and plasma OT levels increased, suggesting that OT induced its own synthesis and release into the bloodstream. This raised the possibility that OT might modulate lipid metabolism in adipose tissue by direct action. This was tested and confirmed using *in vitro* differentiated 3T3-L1 adipocytes, isolated epididymal fat pads studied *ex vivo*, as well as *in vivo* peripheral administration in lean or obese rats.

We next attempted to delineate the mechanisms by which OT stimulated fatty acid β-oxidation in adipose tissue. Both in centrally OT-infused rats, as well as in cultured differentiated 3T3L1 adipocytes, we observed OT-induced increases in eWAT *Scd1* mRNA expression. The importance of Scd1 in metabolic homeostasis was previously evidenced in studies conducted with *Scd1*-deficient mice. Although hyperphagic, these mice were protected against obesity, exhibiting decreased expression of lipogenic enzymes and increased expression of enzymes involved in fatty acid β-oxidation in the liver. In addition, Scd1 deficiency promoted reduced hepatic TG storage [25]. However, the consequences of Scd1 deficiency are complex, as, in addition to the anti-obesity effects just described, other data provide evidence that Scd1 inhibition is detrimental to adipocyte function, leading to unwanted side effects. Such side effects appear to result from increases in the ratio of saturated versus monounsaturated fatty acids in membrane phospholipids (PL) [26], [27], [28], [29]. Of particular interest for our study, decreased membrane oleic acid PL was observed in *Scd1*-deficient mice [28], *Scd1* knockdown in human adipocytes [29] and HeLa cells [27]. According to these results, the OT-induced increase in *Scd1* expression observed in our study was expected to increase the production of oleic acid PL, particularly when considering that lipid storage was unchanged by OT infusion. Oleic acid is known to serve as a precursor of NOPE, leading to the synthesis of OEA, a member of the endocannabinoid-related family of lipids, which was previously shown to reduce food intake [7]. Consistent with our observation of elevated OT-induced *Scd1* expression, we observed increases in eWAT NOPE and OEA content. Interestingly, OEA has been shown to stimulate lipolysis and fatty acid oxidation, effects resulting from OEA activation of PPAR-alpha [23], [24]. These effects may be important during food deprivation, when both plasma OT levels [30] and WAT OEA content [31] are reportedly increased. In addition, most of the OT upregulated genes that encoded enzymes involved in β-oxidation were PPAR-alpha target genes [32], consistent with the hypothesis that OT enhances adipose tissue PPAR-alpha activity. Supporting this notion, we observed that peripheral OT administration promoted a significant decrease in body weight gain and an increase in the expression of genes involved in fatty acid oxidation in wild-type (WT) mice, but had no effect in *PPAR-alpha* knockout animals. The additional observation that OT enhanced *Scd1* expression not only in WT, but also in *PPAR-alpha* knockout mice suggested that the OT effect on *Scd1* was upstream PPAR-alpha activation. This is consistent with the reported downregulation of the PPAR-alpha pathway in the heart of *Scd1*-deficient mice [33], as well as with the upregulation of *Scd1* and the PPAR-alpha pathway in muscles after endurance training [34].

Interestingly, while our results suggest that OT acts on adipose tissue via OEA and PPAR-alpha, a very recent study shows that peripheral OEA suppresses feeding via increasing the central release of OT [35]. This increase was absent in PPAR-alpha KO mice, and the pharmacological blockade of central OT receptors was able to prevent the anorexigenic effects of OEA [35]. These data, together with those of the present study, support the existence of complex interrelationships between OT and OEA, which would play an important role in the regulation of food intake and lipid metabolism.

In addition to protecting rats from HFD-induced obesity, we found that central OT infusion improved glucose tolerance and significantly reduced insulin secretion during the glucose tolerance test, indications of increased insulin sensitivity. Such improvement in insulin sensitivity was confirmed by euglycemic-hyperinsulinemic clamps carried out in both centrally and peripherally OT-infused rats. Regarding the mechanisms underlying such OT effects, they remain to be unraveled. The fact that OEA administration was reported to promote glucose intolerance without altering insulin levels [36] suggests that the OT effects on glucose tolerance are independent from OEA synthesis. Altogether, these results are in keeping with those of a previous study showing that OT-deficient mice exhibit glucose intolerance and decreased insulin sensitivity [4].

In view of the present data, the question of the metabolic impact of physiological OT increases, such as those occurring during labor and lactation can be raised. Although speculative, some hypothesis can be proposed and might deserve to be tested. During pregnancy, central opioids transiently inhibit OT neurons to prevent preterm labor [37]. This inhibits OT release in the posterior pituitary and lowers plasma OT levels [37]. Knowing that about 3–15% of women develop gestational diabetes mellitus [38], characterized by insulin resistance and increased insulin secretion, our results could suggest that inhibition of OT neurons would, at least partly, be responsible for this insulin resistant state. Conversely, during suckling, OT is secreted to promote milk ejection. Potentially, the elevation in OT levels during this period could contribute to the increased Lpl activity observed in the mammary gland [39].

Considering a potential use of OT as an anti-obesity agent, an important issue to be addressed is that of potential side effects of the treatment. As eluded to above, one of the main physiological OT effects is to induce uterine contraction during labor, a condition in which plasma OT levels increase by 7 folds [40]. One of the potential side effects of an anti-obesity OT treatment would therefore be to promote uterine contraction. However, during labor, it was demonstrated that the OTR concentration in the myometrium is increased by more than 150 times to increase the sensitivity of the uterus to OT [41]. Importantly, it was demonstrated that both the decline of progesterone resulting from luteolysis and the increased endogenous oestrogen levels after luteolysis are necessary for the induction of OTR mRNA during parturition [42]. Taken together, these results suggest that in normal conditions, in which oestrogen levels are normal, basal uterine OTR levels will not allow OT to induce uterine contraction.

In summary, our study confirms and extends the recent data showing anti-obesity effects of central OT administration. It allows concluding that these effects are independent from the anorexigenic action of OT and bear on fat mass loss. The mechanisms involved comprise OEA production in adipose tissue, activation of PPAR-alpha and resulting increased fatty acid β-oxidation. These effects are exerted by direct action of OT on adipocytes. Upon central OT infusion, this is due to the positive feedback effect of the hormone on its own secretion, resulting in dose-dependent increases in plasma OT levels. Similar results can be obtained by peripheral OT administration. Whether infused centrally or peripherally, chronic OT infusion also improves insulin sensitivity of diet-induced obese rats. Altogether, these results suggest that activation of the OT receptor pathway by infusion of OT, OT analogs, or OT agonists represents a promising approach for treating obesity and type 2 diabetes.

## Supporting Information

Figure S1 **Central OT infusion does not modify lipid metabolism in skeletal muscle and in the liver.** mRNA expression of enzymes related to lipid metabolism in: (A) quadriceps and (B) the liver of saline–infused controls (filled bars) and i.c.v. oxytocin-infused rats (1.6 nmol/d; open bars). Values are mean ± SEM of 6 to 7 animals/group. Intergroup differences: NS.
(TIF)

Figure S2 **Central OT infusion does not modify OT and OTR mRNA expression in eWAT.** The following parameters were measured at the end of 14-day treatments with two doses of i.c.v. OT infusion: (A) Oxytocin (*Oxt*) and (B) Oxytocin receptor (*OxtR*) expression in rat eWAT of saline–infused controls (filled bars) and OT-infused rats (1.6 nmol/d, open bars). Values are mean ± SEM of 6 to 7 rats/group. (C) Oxytocin (*Oxt*) and (D) Oxytocin receptor (*OxtR*) expression in rat eWAT of saline–infused controls (filled bars), OT-infused rats (16 nmol/d, open bars) and pair-fed (PF) controls (hatched bars). Values are mean ± SEM of 6 to 7 rats/group.
(TIF)

Figure S3 **OEA affects lipid metabolism in cultured adipocytes.** (A) PPAR-alpha and PPAR-alpha target gene expression in differentiated 3T3-L1 adipocytes (24 h vehicle or 0.2 µM OEA). Values are mean ± SEM of three independent experiments. *P<0.05 compared to controls.
(TIF)

Figure S4 **Effects of peripheral OT infusion on lipid metabolism-related parameters in eWAT.** The following analyses were performed on eWAT of s.c. saline–infused controls (filled bars), s.c. OT-infused rats (50 nmol/d; open bars), and s.c. saline-infused PF controls (hatched bars): (A) TG; (B) FFA and (C) glycerol content. Values are mean ± SEM of 7 to 8 rats/group. *P<0.05, **P<0.01 compared to controls.
(TIF)

Table S1 **Primer sequences used for qPCR.**
(DOC)

Table S2 **Effects of i.c.v. oxytocin (1.6 nmol/d) infusion on food intake, meal number, meal size, meal duration, feeding rate, intermeal interval (IMI) and satiety ratio.** Values are mean ± SEM of 6 animals per group. P = NS for all comparisons.
(DOC)

Table S3 **Effects of i.c.v. oxytocin (16 nmol/d) infusion on food intake, meal number, meal size, meal duration, feeding rate, intermeal interval (IMI) and satiety ratio.** Values are mean ± SEM of 7 animals per group. * P<0.05, ** P<0.01 versus saline-infused controls. P = NS for all other comparisons.
(DOC)
