# Supplementary figures and images for: Mechanisms of the Anti-Obesity Effects of Oxytocin in Diet-Induced Obese Rats
Source: PLoS One. 2011 Sep 27;6(9):e25565. doi: 10.1371/journal.pone.0025565 (PMC3181274; doi:10.1371/journal.pone.0025565)

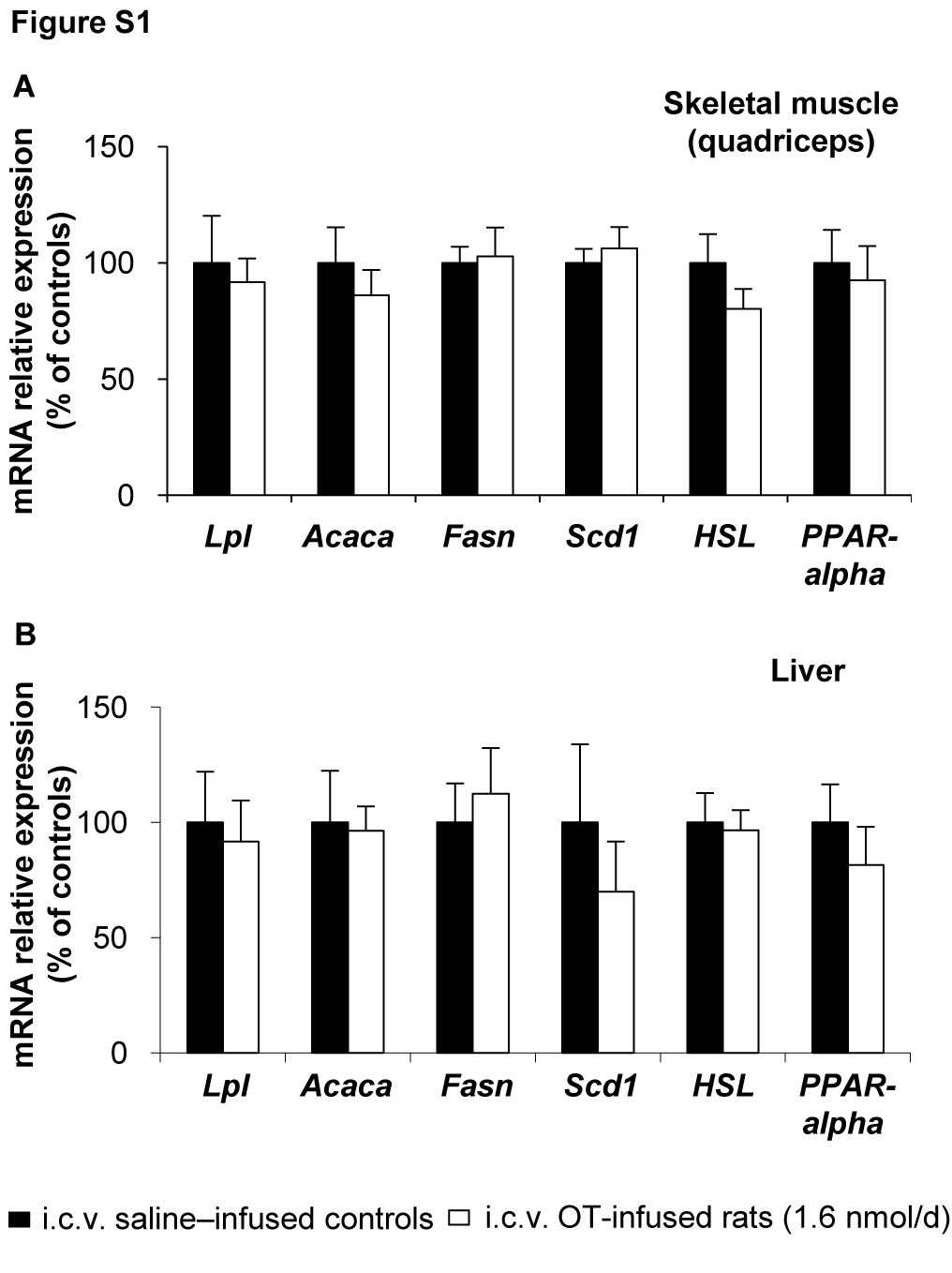

Supplement: Figure S1 — Central OT infusion does not modify lipid metabolism in skeletal muscle and in the liver. mRNA expression of enzymes related to lipid metabolism in: (A) quadriceps and (B) the liver of saline–infused controls (filled bars) and i.c.v. oxytocin-infused rats (1.6 nmol/d; open bars). Values are mean ± SEM of 6 to 7 animals/group. Intergroup differences: NS. (TIF) [file pone.0025565.s001.tif]

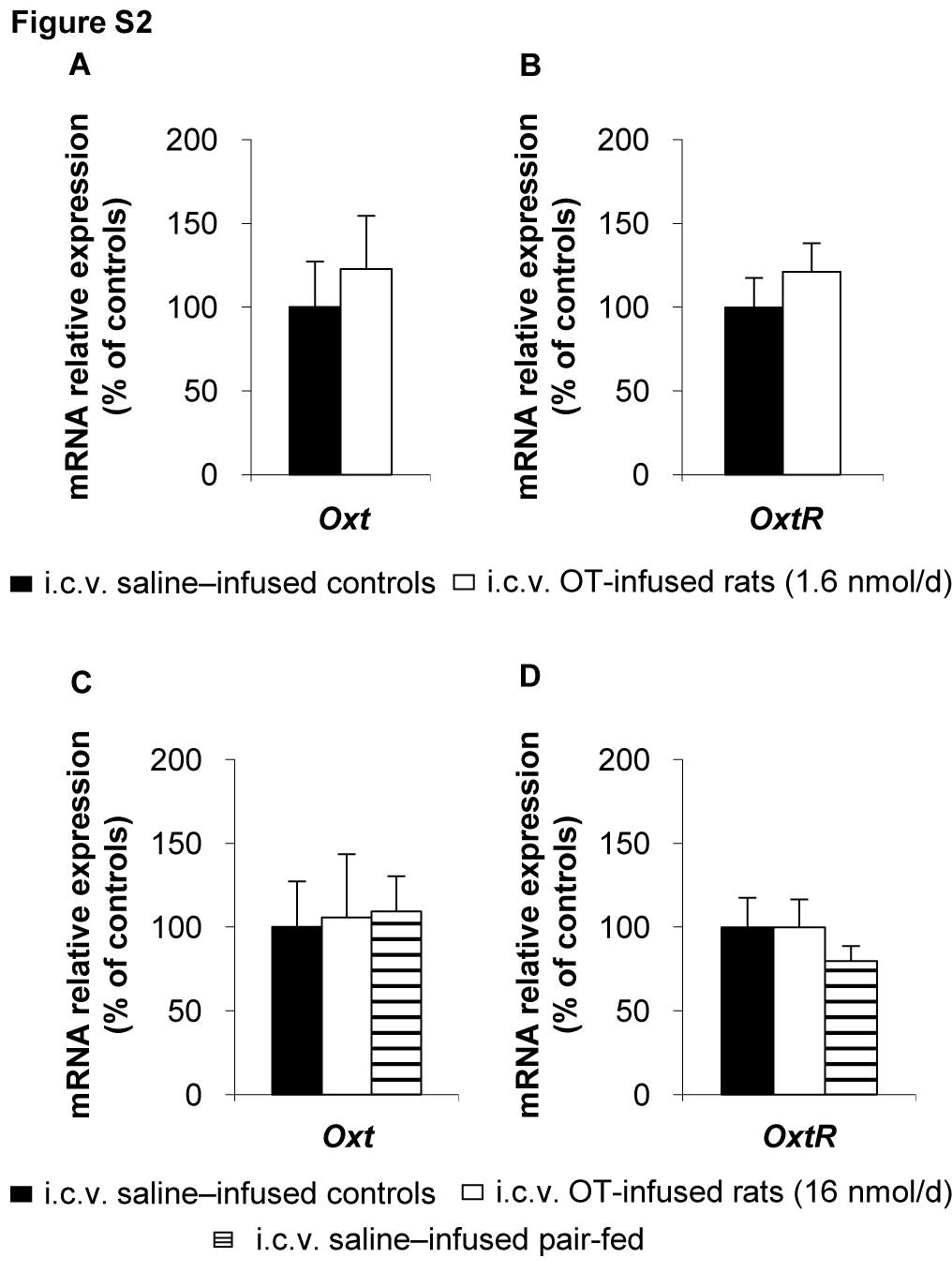

Supplement: Figure S2 — Central OT infusion does not modify OT and OTR mRNA expression in eWAT. The following parameters were measured at the end of 14-day treatments with two doses of i.c.v. OT infusion: (A) Oxytocin (Oxt) and (B) Oxytocin receptor (OxtR) expression in rat eWAT of saline–infused controls (filled bars) and OT-infused rats (1.6 nmol/d, open bars). Values are mean ± SEM of 6 to 7 rats/group. (C) Oxytocin (Oxt) and (D) Oxytocin receptor (OxtR) expression in rat eWAT of saline–infused controls (filled bars), OT-infused rats (16 nmol/d, open bars) and pair-fed (PF) controls (hatched bars). Values are mean ± SEM of 6 to 7 rats/group. (TIF) [file pone.0025565.s002.tif]

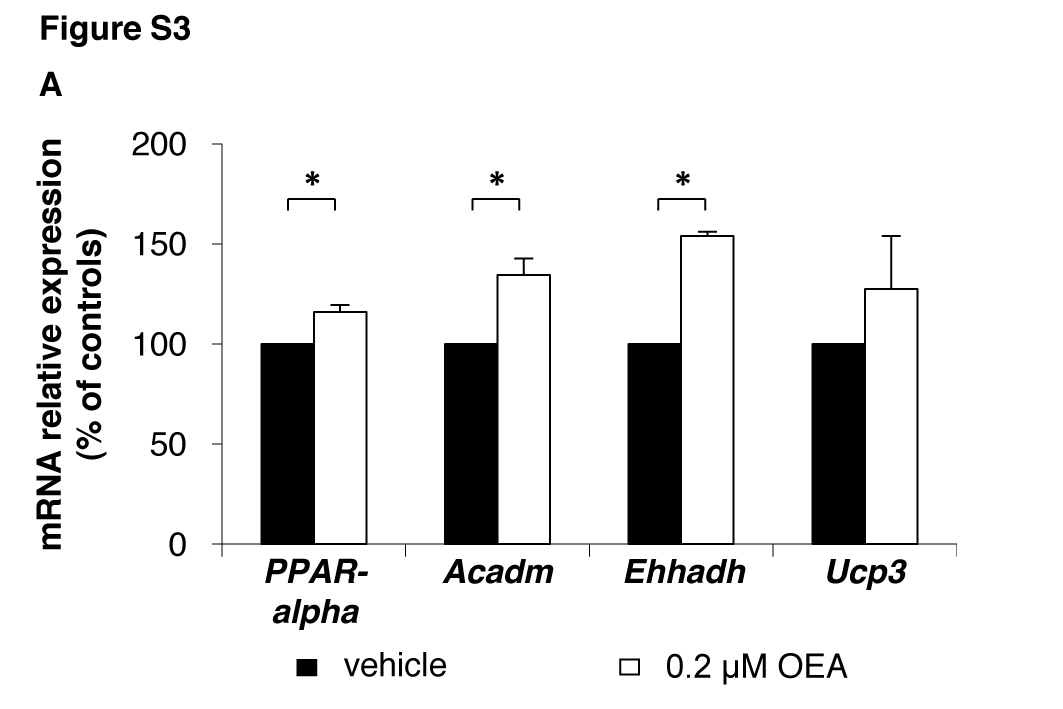

Supplement: Figure S3 — OEA affects lipid metabolism in cultured adipocytes. (A) PPAR-alpha and PPAR-alpha target gene expression in differentiated 3T3-L1 adipocytes (24 h vehicle or 0.2 µM OEA). Values are mean ± SEM of three independent experiments. *P<0.05 compared to controls. (TIF) [file pone.0025565.s003.tif]

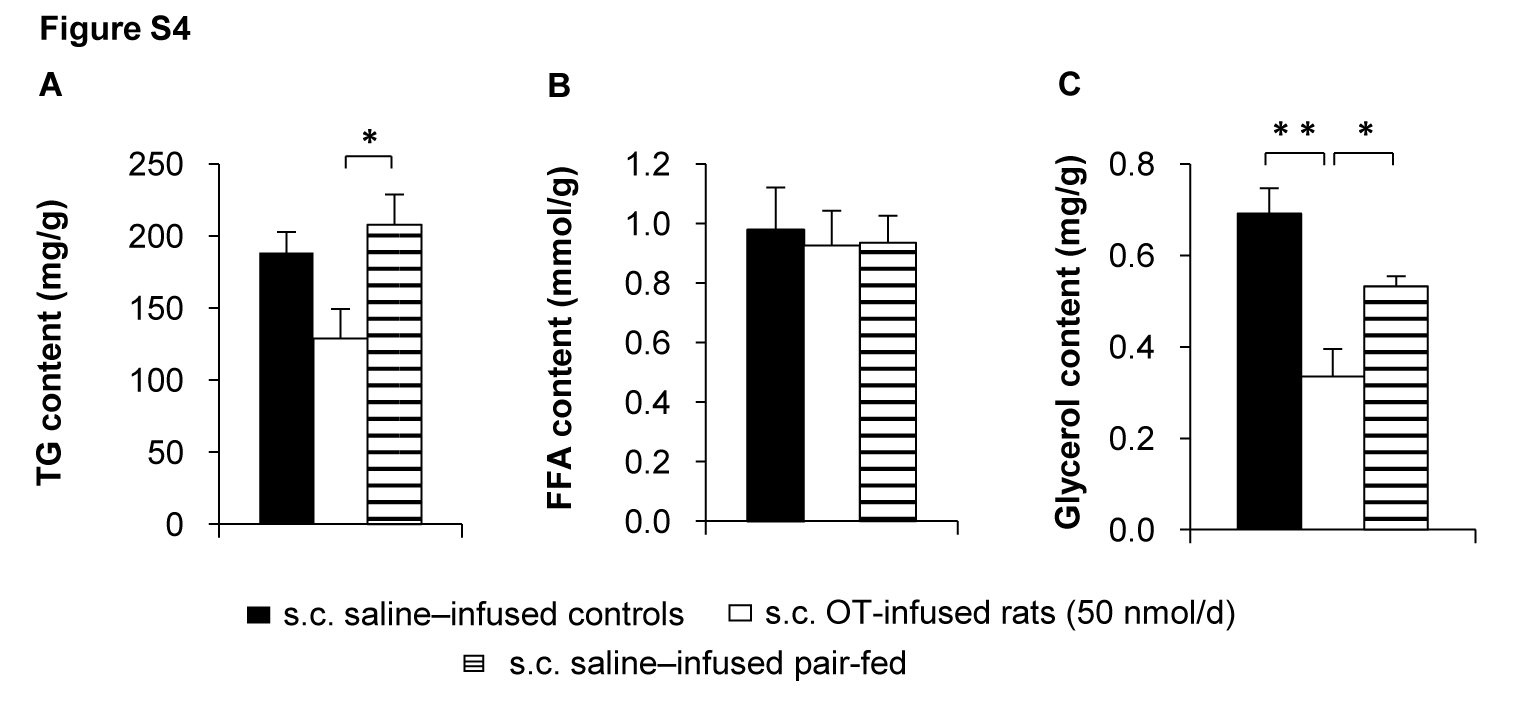

Supplement: Figure S4 — Effects of peripheral OT infusion on lipid metabolism-related parameters in eWAT. The following analyses were performed on eWAT of s.c. saline–infused controls (filled bars), s.c. OT-infused rats (50 nmol/d; open bars), and s.c. saline-infused PF controls (hatched bars): (A) TG; (B) FFA and (C) glycerol content. Values are mean ± SEM of 7 to 8 rats/group. *P<0.05, **P<0.01 compared to controls. (TIF) [file pone.0025565.s004.tif]
