# Supplementary material for: Mechanisms of the Anti-Obesity Effects of Oxytocin in Diet-Induced Obese Rats
Source: PLoS One. 2011 Sep 27;6(9):e25565. doi: 10.1371/journal.pone.0025565 (PMC3181274; doi:10.1371/journal.pone.0025565)
Supplement: Table S1 — Primer sequences used for qPCR. (DOC) [file pone.0025565.s005.doc]

**Table S1**

| **Gene** | **Ascension Number** | **Forward primer sequence** | **Reverse primer sequence** |
| --- | --- | --- | --- |
| *Lpl* | L03294 | 5’-GGCATACAGGTGCAATTCCA | 5’-CGTCGAACTTGGACAGATCCTT |
| *Fat* | NM001109218.1 | 5’-AGTAATCTCAAATAACTGTACGTCG | 5’-CTGCAAGCACAGTATGAAATCATAA |
| *Acaca* | J03808 | 5’-ACCTCAACCACTACGGCATGA | 5’-AGGTGGTGTGAAGGCGTTGT |
| *Fasn* | NM017332 | 5’-GGACATGGTCACAGACGATGAC | 5’-CGTCGAACTTGGACAGATCCTT |
| *Dgat1* | NM053437 | 5’-GTTCAGCTCAGACAGCGGTTT | 5’-CATCACCACGCACCAATTCA |
| *Pnpla2* | NM001108509 | 5’-GGCCATGATGGTGCCCTATA | 5’-CCAACAAGCGGATGGTGAA |
| *Hsl* | NM012859 | 5’-CCCCGAGATGTCACAGTCAAT | 5’-GAATTCCCGGATCGCAGAA |
| *PPAR-alpha* | NM013196 | 5’-GTCCCTCGGAGAGG | 5’-GGAAGCTGGAGAGA |
| *Acox1* | NM0173340 | 5’-CGACCTTGTTCGGGCAAGT | 5’-TGAGAAGACCTTAACGACCACGTA |
| *Ehhadh* | NM133606 | 5’-TCCCTGGCTTTCTACGTTCCT | 5’-GATGGTGCGCTGCTCGAT |
| *Acadm* | NM016986 | 5’- CGCCGGAACACGTACTTTG | 5’-CGAGCTGGTTGGCAATATCTC |
| *Ucp3* | NM019354 | 5’-GGACAGCAGCCTGTATTGCA | 5’-GGGTTGCACTTCGGAAGTTGT |
| *Scd1* | NM139192 | 5’-CCGTGGCTTTTTCTTCTCTCA | 5’-CTTTCCGCCCTTCTCTTTGA |
| *Oxt* | NM012996 | 5’-CGCCTGCGACCCTGAGT | 5’-AAGGAAGCGCCCTAAAGGTATC |
| *OxtR* | NM012871.2 | 5’-CATCACCTTCCGCTTCTATGG | 5’-ATGCCCACCACCTGCAAGTA |
