# Supplementary material for: Mechanisms of the Anti-Obesity Effects of Oxytocin in Diet-Induced Obese Rats
Source: PLoS One. 2011 Sep 27;6(9):e25565. doi: 10.1371/journal.pone.0025565 (PMC3181274; doi:10.1371/journal.pone.0025565)
Supplement: Table S2 — Effects of i.c.v. oxytocin (1.6 nmol/d) infusion on food intake, meal number, meal size, meal duration, feeding rate, intermeal interval (IMI) and satiety ratio. Values are mean ± SEM of 6 animals per group. P = NS for all comparisons. (DOC) [file pone.0025565.s006.doc]

**Table S2**

|  | **Saline-infused rats** | **OT-infused rats** | T test |
| --- | --- | --- | --- |
|  |  |  |  |
| **Food intake (g/d)** |  |  |  |
| Total | 17.2 ± 0.7 | 16.0 ± 1.2 | 0.418 |
| Nocturnal | 12.5 ± 0.8 | 11.6 ± 1.4 | 0.626 |
| Diurnal | 4.8 ± 0.5 | 4.4 ± 0.8 | 0.670 |
|  |  |  |  |
| **Meal number**  **(meals/d)** |  |  |  |
| Total | 11.8 ± 0.5 | 10.8 ± 0.3 | 0.140 |
| Nocturnal | 8.0 ± 0.7 | 7.2 ± 0.5 | 0.341 |
| Diurnal | 3.8 ± 0.5 | 3.7 ± 0.3 | 0.780 |
|  |  |  |  |
| **Meal size (g/d)** |  |  |  |
| Total | 1.5 ± 0.2 | 1.5 ± 0.2 | 0.888 |
| Nocturnal | 1.6 ± 0.2 | 1.7 ± 0.3 | 0.818 |
| Diurnal | 1.5 ± 0.3 | 1.4 ± 0.1 | 0.778 |
|  |  |  |  |
| **Meal duration (min)** |  |  |  |
| Total | 1.7 ± 0.3 | 1.2 ± 0.2 | 0.209 |
| Nocturnal | 1.5 ± 0.3 | 1.2 ± 0.2 | 0.401 |
| Diurnal | 2.2 ± 1.2 | 1.2 ± 0.2 | 0.337 |
|  |  |  |  |
| **Feeding rate (g/min)** |  |  |  |
| Total | 1.4 ± 0.2 | 1.5 ± 0.2 | 0.600 |
| Nocturnal | 1.5 ± 0.2 | 1.6 ± 0.3 | 0.545 |
| Diurnal | 1.3 ± 0.3 | 1.4 ± 0.1 | 0.851 |
|  |  |  |  |
| **IMI (min)** |  |  |  |
| Total | 116.5 ± 7.8 | 126.7 ± 3.9 | 0.271 |
| Nocturnal | 106.7 ± 7.5 | 116.0 ± 11.8 | 0.519 |
| Diurnal | 152.3 ± 15.7 | 181.5 ± 21.0 | 0.292 |
|  |  |  |  |
| **Satiety ratio (min/g)** |  |  |  |
| Total | 79.3 ± 5.2 | 87.1 ± 8.9 | 0.466 |
| Nocturnal | 69.7 ± 4.9 | 75.1 ± 8.7 | 0.607 |
| Diurnal | 114.7 ± 14.7 | 135.5 ± 16.4 | 0.365 |
