# Supplementary material for: Mechanisms of the Anti-Obesity Effects of Oxytocin in Diet-Induced Obese Rats
Source: PLoS One. 2011 Sep 27;6(9):e25565. doi: 10.1371/journal.pone.0025565 (PMC3181274; doi:10.1371/journal.pone.0025565)
Supplement: Table S3 — Effects of i.c.v. oxytocin (16 nmol/d) infusion on food intake, meal number, meal size, meal duration, feeding rate, intermeal interval (IMI) and satiety ratio. Values are mean ± SEM of 7 animals per group. * P<0.05, ** P<0.01 versus saline-infused controls. P = NS for all other comparisons. (DOC) [file pone.0025565.s007.doc]

**Table S3**

|  | **Saline-infused rats** | **OT-infused rats** | T test |
| --- | --- | --- | --- |
|  |  |  |  |
| **Food intake (g/d)** |  |  |  |
| Total | 17.6 ± 0.7 | 14.5 ± 0.6****** | **0.005** |
| Nocturnal | 12.4 ± 0.6 | 10.6 ± 1.0 | 0.143 |
| Diurnal | 5.1 ± 0.6 | 3.9 ± 0.6 | 0.159 |
|  |  |  |  |
| **Meal number**  **(meals/d)** |  |  |  |
| Total | 12.6 ± 0.9 | 9.7 ± 0.5***** | **0.014** |
| Nocturnal | 8.7 ± 0.9 | 7.0 ± 0.7 | 0.168 |
| Diurnal | 3.9 ± 0.4 | 2.7 ± 0.5 | 0.091 |
|  |  |  |  |
| **Meal size (g/d)** |  |  |  |
| Total | 1.5 ± 0.1 | 1.6 ± 0.1 | 0.581 |
| Nocturnal | 1.5 ± 0.2 | 1.6 ± 0.1 | 0.886 |
| Diurnal | 1.5 ± 0.2 | 1.8 ± 0.2 | 0.401 |
|  |  |  |  |
| **Meal duration (min)** |  |  |  |
| Total | 1.7 ± 0.3 | 1.4 ± 0.2 | 0.430 |
| Nocturnal | 1.4 ± 0.3 | 1.4 ± 0.2 | 1.000 |
| Diurnal | 2.7 ± 1.0 | 1.1 ± 0.1 | 0.160 |
|  |  |  |  |
| **Feeding rate (g/min)** |  |  |  |
| Total | 1.3 ± 0.1 | 1.5 ± 0.1 | 0.456 |
| Nocturnal | 1.4 ± 0.2 | 1.5 ± 0.1 | 0.720 |
| Diurnal | 1.3 ± 0.3 | 1.7 ± 0.3 | 0.498 |
|  |  |  |  |
| **IMI (min)** |  |  |  |
| Total | 111.6 ± 8.2 | 145.6 ± 7.9***** | **0.012** |
| Nocturnal | 100.9 ± 8.6 | 136.3 ± 5.6****** | **0.005** |
| Diurnal | 152.7 ± 13.3 | 182.6 ± 16.9 | 0.222 |
|  |  |  |  |
| **Satiety ratio (min/g)** |  |  |  |
| Total | 77.5 ± 4.7 | 93.5 ± 2.6***** | **0.012** |
| Nocturnal | 68.3 ± 4.4 | 89.8 ± 5.3****** | **0.009** |
| Diurnal | 110.6 ± 13.0 | 114.4 ± 16.0 | 0.856 |
